# Rising contraceptive use, falling couple concordance: A mixed-methods analysis of the concordance paradox in Nigeria

**DOI:** 10.1371/journal.pgph.0007450

**Published:** 2026-10-30

**Authors:** Olaniyi Felix Sanni, Abike Elizabeth Sanni, Collins Isioma Onyeagwaibe

**Affiliations:** Research and Development Department, Fescosof Data Solutions, Ogun State, Nigeria; Hamadan University of Medical Sciences School of Public Health, IRAN, ISLAMIC REPUBLIC OF

## Abstract

Couple agreement on contraception is widely treated as a marker of programme success, yet whether such agreement has kept pace with rising contraceptive use in sub-Saharan Africa is largely unknown. We analysed three rounds of the Nigeria Demographic and Health Survey Couples’ Recode (2013, 2018, 2024; 23,547 couples) using design-weighted descriptive, trend, and multivariable logistic analyses. We defined practice concordance from partners’ independently reported current method status and decomposed it into five mutually exclusive categories. A qualitative study of 42 Ogun State couples illustrated the mechanism behind the pattern. National practice concordance fell from 81.6% to 73.5% (and from 70.0% to 46.4% in Ogun State) even as women's modern use (9.7% to 16.1%), spousal communication (33.0% to 42.1%), and Unmet need (16.1% to 21.3%) rose. Concordance was overwhelmingly shared non-use (69.5% in 2024); genuine mutual active use remained near 4%; and wife-only discordance nearly doubled (7.5% to 14.0%), which supplied most of the offsetting rise in discordance as shared non-use contracted. An inverse socioeconomic gradient and a north-south gradient persisted after adjustment, and spousal communication was negatively associated with concordance (odds ratio 0.83, 95% CI 0.76–0.91). Rising, increasingly female-led contraceptive practice is associated at the population level with falling couple concordance, a pattern consistent with an incomplete gender transition rather than programme failure.

## Introduction

Couple agreement on contraceptive practice has long been read as a sign that family planning programmes are working. When partners report the same method status, the inference is that communication is open, decisions are shared, and a wanted method is being used without friction. Concordance has accordingly become a familiar outcome in the couples literature, treated as a near-synonym for programme success and for the kind of joint decision-making that fertility-reduction efforts are meant to foster [1,2]. Yet the inference rests on an assumption that is rarely examined: that agreement and contraceptive uptake move together, so that as use rises couples increasingly align on a shared, active method.

That assumption deserves scrutiny because agreement is not one thing. Two partners can agree because both uses modern method, or because neither is using anything at all. The first is the active, jointly protected state the literature has in mind; the second is a shared absence of use that registers as agreement on any measure built from matched method status. Where contraceptive prevalence is low, shared non-use dominates the agreement category by sheer arithmetic, and a population can post high concordance while almost no couples are jointly protected. Agreement, in other words, can capture either coordinated action or coordinated inaction, and a single summary figure does not separate them.

Nigeria is an instructive setting for this question. Modern contraceptive prevalence remains low by regional standards but has risen over the past decade, spousal communication about family planning has become more common, and women's use has grown faster than men's, increasingly led by women [3,4]. The country also retains a pronounced north-south gradient in reproductive behaviour and strongly gendered norms around fertility decision-making [5,6]. These features make it possible, in principle, for rising and increasingly female-led use to pull partners apart rather than together, at least as captured by matched method reports.

The couples literature has not been well positioned to detect such a divergence. Most studies analyse a single survey round and treat agreement as a static trait of couples rather than a quantity that can move over time [1,7]. Multi-round, couple-level trend analysis is scarce, and the internal composition of the agreement category, in particular the share that is shared non-use as against genuine mutual use, is rarely reported. As a result, it is unknown whether couple concordance in sub-Saharan Africa has kept pace with the contraceptive gains of the past decade, or whether headline agreement figures conceal a shift in what agreement is made of.

We address that gap using three rounds of the Nigeria Demographic and Health Survey Couples’ Recode (2013, 2018, 2024), the largest nationally representative couple dataset available for the country. We define practice concordance from partners’ independently reported current method status, track it across the three rounds at national and state level, and decompose it into five mutually exclusive categories so that shared non-use, mutual active use, and the discordant states can be examined separately. We aimto establish whether couple concordance has risen or fallen alongside contraceptive use in Nigeria between 2013 and 2024, and to characterise what that movement reveals about the contraceptive transition underway. We read the findings through the Theory of Planned Behaviour [8], the gender-and-power framework [9,10], and the social-ecological model [11], developed in the Discussion.

## Materials and methods

### Data and study sample

We used the Couples’ Recode of the Nigeria Demographic and Health Survey for 2013, 2018, and 2024 (the latest round was fielded in 2023–24 and is referred to throughout as 2024). The analysis used the released 2024 Couples’ Recode dataset associated with the final 2024 NDHS; it was therefore not based on preliminary survey estimates. The Couples’ Recode links the records of co-resident married or cohabiting partners who were each interviewed independently, allowing method status to be compared partner to partner without one spouse reporting on the other. Pooling the three rounds yielded 23,547 couples (8,658 in 2013; 8,061 in 2018; 6,828 in 2024). Current method status was available for both partners in all 23,547 couples; the multivariable analytic sample was 23,444 complete cases (99.6% of the linked total), the excluded 0.4% lacking a valid response on the spousal-communication item. Current contraceptive method was taken from variable V312 for women and MV312 for men.

### Outcome: practice concordance and its decomposition

Our primary outcome was practice concordance, defined from partners’ independently reported current contraceptive use status. A couple was classified as concordant when both partners reported the same practice status and discordant otherwise. This use-status definition was selected to capture whether contraceptive practice was shared within the couple, irrespective of whether partners reported the same specific method. We decomposed concordance and discordance into five mutually exclusive categories: shared non-use (neither partner using any method); mutual active use (both partners reporting current contraceptive use); wife-only use (the woman reports use and the man does not); husband-only use (the man reports use and the woman does not); and use-discordant couples in which both partners report use but different methods.

The five categories partition every couple exactly once, so their shares sum across the population and movements between them can be read directly.

We treat shared non-use as a genuine agreement state rather than excluding it, and we want to be explicit about why. Agreement measures built from matched method reports count shared non-use as concordance whether or not the analyst names it; removing it would not make the category disappear from the headline figure, it would only hide the mechanism. Retaining it and reporting it separately is what allows the central question, whether rising use is accompanied by rising joint protection, to be answered. The decomposition is also the test of our own interpretation: if the fall in concordance were an artefact of how agreement is defined, it would not survive being broken into components. It does survive, because the movement is concentrated in specific, substantively meaningful cells (shared non-use contracting as wife-only use expands), not spread diffusely across the partition.

### Survey design and estimation

All estimates account for the complex survey design. We applied the women's sample weight (V005 divided by 1,000,000), specified sampling strata (V022) and primary sampling units (V021), and used Taylor-series linearization with PSU cluster-robust standard errors. Because DHS weights are normalised within each survey round, the pooled models include survey-round indicators, and the round-stratified models use each round’s own weights. Analyses were conducted in Python using pandas and statsmodels for data management and modelling, samplics for design-based estimation, and geopandas for the geographic work.

### Statistical analysis

We first described the sample by survey round and summarised the key indicators (practice concordance, women's modern method use, spousal communication about family planning, and unmet need) for each round at national level and for Ogun State. Trends across rounds were assessed with design-based Wald tests, the survey-weighted analogue of the Pearson chi-square test, and we report the direction, magnitude, and significance of change rather than treating any single round in isolation. We then examined the five-category decomposition over time to locate the source of any change in concordance.

Correlates of concordance were examined first bivariately and then in a multivariable logistic regression with concordance as the binary outcome. The adjusted model included place of residence, education, household wealth, educational homogamy between partners, age, and parity, alongside spousal communication. All models used the survey design specification above, and we report adjusted odds ratios with cluster-robust confidence intervals. Educational homogamy, coded as a couple-level indicator of equal educational attainment between partners, was included both as a covariate and as a substantive question, because shared educational standing is often assumed to promote agreement. Diagnostic results were reviewed alongside coefficient estimates and confidence intervals to assess overall model performance. Because the analyses accounted for the complex survey design, diagnostics were interpreted in the context of the survey-weighted estimation framework.

For the geographic analysis, we mapped state-level concordance and summarised the national gradient, retaining the design weights so that state estimates are population-representative. Because state-level cells are smaller, we read these estimates as descriptive of spatial pattern rather than as precise point values for any single state. Reporting of the quantitative strand follows the STROBE (Strengthening the Reporting of Observational Studies in Epidemiology) recommendations for cross-sectional studies [12]; the completed checklist is provided in S1 File.

### Qualitative corroboration

To corroborate and explain the population-level pattern, we drew on a qualitative study of 42 couples (84 individuals) purposively recruited from five local government areas of Ogun State (Ado-Odo/Ota, 10 couples; Abeokuta South, Ifo, Sagamu, and Yewa South, 8 couples each). Recruitment sought couples with experience of family-planning decision-making and variation in reported contraceptive practice, while the qualitative component was intended to illuminate mechanisms rather than estimate prevalence.

Ogun State was purposively selected because the quantitative analysis showed a particularly pronounced decline in concordance alongside relatively high female contraceptive use and spousal communication, making it a useful setting for examining the mechanisms underlying the national pattern. Interviews were conducted between March and April 2026. Each couple participated in a joint interview followed by separate individual interviews with each partner, allowing both shared and potentially divergent perspectives to be explored while reducing the likelihood that one partner's responses constrained the other's account.

Interviewers were trained before data collection on the study objectives, interview guide, dyadic interviewing procedures, informed consent, confidentiality, neutrality, and management of potentially sensitive discussions about contraceptive decision-making. Interviews followed a semi-structured guide and were conducted in a manner that encouraged participants to describe contraceptive communication, decision-making, initiation of methods, and experiences of agreement or disagreement with their partners. Interviews were audio-recorded with participants’ permission, transcribed verbatim, and checked against the recordings for completeness and accuracy.

Data were analysed thematically following Braun and Clarke [13]. The analysis involved repeated reading of transcripts, generation of initial codes, grouping related codes into candidate themes, review and refinement of themes against the coded extracts and full dataset, and definition and naming of the final themes. Coding focused on processes relevant to the quantitative findings, including female-led information-seeking and method initiation, spousal communication, negotiation of desired family size, independent or covert contraceptive use, and movement between shared non-use and active use. The analysis was iterative, with emerging interpretations compared across joint and individual interviews and across couples to identify convergence and divergence.

Analytic rigor was supported through systematic use of the interview guide and coding framework, comparison of accounts from both partners, repeated review of transcripts and coded material, and discussion of emerging interpretations among the research team. The qualitative findings were also considered alongside the quantitative decomposition to assess whether the reported experiences provided a plausible explanation for the observed population-level pattern. Reporting was structured against the COREQ checklist [14]. The qualitative sample was not intended to reproduce the prevalence of wife-only discordance observed nationally; rather, it served as a mechanism-oriented component illustrating how female-led and, in some cases, independent or covert contraceptive adoption can arise within couples.

### Ethics

This study analysed two data sources. The quantitative strand used the publicly available Demographic and Health Survey Couples’ Recode files for Nigeria. The DHS Program obtains informed consent from all participants and operates under ethical clearance for each survey round; the datasets were de-identified, contain no names or household addresses, and carry no geographic identifiers below the regional level. Authorisation to use the Nigeria DHS datasets for this registered project was granted by The DHS Program on 16 March 2026. The qualitative strand was approved by the Institutional Review Board of the Nigerian Institute of Medical Research, Yaba, Lagos (project number IRB/26/008; approved 26 February 2026, valid to 25 February 2027). Written informed consent was obtained from each partner independently; where a participant could not read, the consent form was read aloud in the presence of an impartial witness and a thumbprint consent was recorded.

## Results

### Sample characteristics

The three rounds contributed 8,658 couples in 2013, 8,061 in 2018, and 6,828 in 2024, for a pooled total of 23,547 (Table 1). Method status was available for both partners in all 23,547 couples, so missingness on the outcome was negligible and unlikely to bias the trend estimates; the multivariable models used 23,444 complete cases (99.6%), the small excluded fraction concentrated in the spousal-communication item. All figures that follow are design-weighted and therefore representative of married and cohabiting couples nationally, with Ogun State reported separately as a focal case.

**Table 1:** Weighted demographic characteristics of couples by survey round, Nigeria DHS 2013, 2018, and 2023–24.

Characteristic	2013	2018	2024
Total couples, N (unweighted)	8,658	8,061	6,828
Women's age, mean ± SD	28.3 ± 7.3	30.9 ± 7.9	31.1 ± 7.9
Men's age, mean ± SD	36.7 ± 7.4	39.8 ± 8.8	40.5 ± 8.6
Residence, Urban, %	34.0	42.6	41.6
Residence, Rural, %	66.0	57.4	58.4
Women's education, No education, %	48.6	41.5	42.0
Women's education, Primary, %	19.0	15.8	12.8
Women's education, Secondary, %	25.4	32.9	32.5
Women's education, Higher, %	7.0	9.9	12.7
Men's education, No education, %	34.5	30.2	32.1
Men's education, Primary, %	21.5	17.4	13.4
Men's education, Secondary, %	30.6	35.6	33.6
Men's education, Higher, %	13.4	16.8	20.8
Wealth, Poorest, %	24.3	19.0	20.3
Wealth, Poorer, %	21.7	20.1	20.4
Wealth, Middle, %	17.2	19.8	18.9
Wealth, Richer, %	17.5	19.6	20.1
Wealth, Richest, %	19.4	21.4	20.4
Living children, mean ± SD	2.9 ± 2.1	3.4 ± 2.2	3.3 ± 2.2
Educational homogamy, %	58.5	59.4	58.7

Note. All cells design-weighted; N denominators unweighted. SD = standard deviation.

### The concordance paradox

The central finding is a divergence between contraceptive practice and couple agreement. Over the period, women's modern method use rose from 9.7% to 14.4% to 16.1%, spousal communication about family planning rose from 33.0% to 39.0% to 42.1%, and unmet need rose from 16.1% to 17.7% to 21.3%. Every one of these indicators moved upward, though rising unmet need signals demand outpacing uptake rather than programme success. Practice concordance moved in the opposite direction, falling nationally from 81.6% in 2013 to 75.1% in 2018 and 73.5% in 2024, a decline of about eight percentage points that was statistically significant (design-based Wald χ² [2 df] = 30.1, p < 0.001); the 2013 and 2024 estimates are precise and non-overlapping (81.6%, 95% CI 79.3–83.6 vs 73.5%, 71.7–75.2). In Ogun State the same divergence was sharper: concordance fell from 70.0% to 60.9% in 2018 and to 46.4% in 2024, a drop of 23.6 points, while spousal communication in the state reached 56.1% by 2024. Rising use and rising communication were thus accompanied by falling agreement, not by the convergence the standard reading would predict. Fig 1 plots these divergent trends; each of the four trends is statistically significant on design-based Wald tests (all p < 0.001).

**Fig 1:**
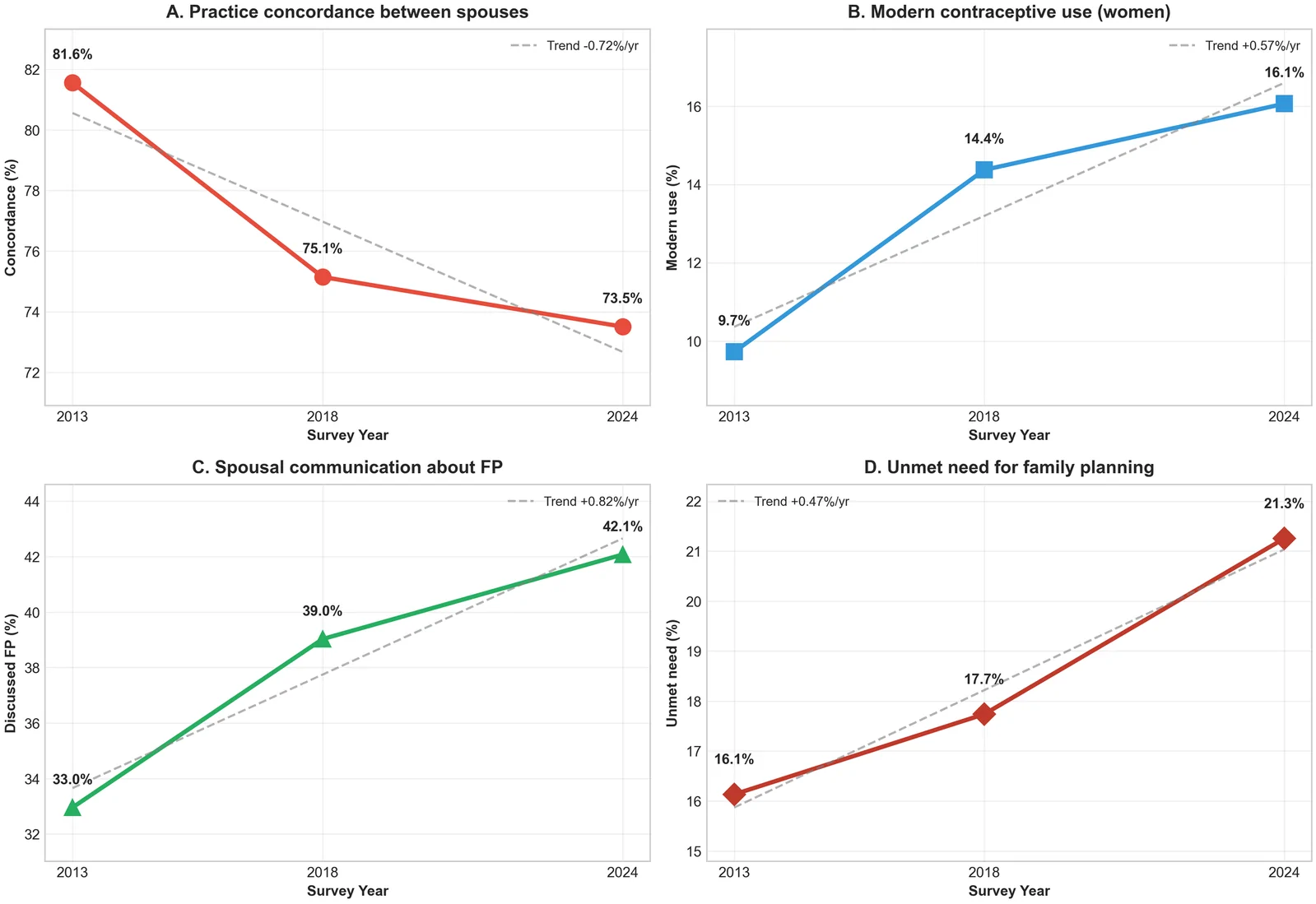
Temporal trends in couple concordance and family planning indicators, Nigeria, 2013 to 2024 (design-weighted).

### Inside the agreement category: the five-category decomposition

Decomposing concordance shows that the agreement being recorded was almost entirely a shared absence of use. In 2024, shared non-use accounted for 69.5% of all couples, while genuine mutual active use, both partners using a method, accounted for only about 4.0%, little different from the 4.1% recorded in 2013 after dipping to 2.5% in 2018. Couple agreement in Nigeria therefore meant, in the great majority of cases, that neither partner was doing anything, and the small pocket of jointly protected couples did not grow as national use rose. The fall in concordance was driven by a specific cell: wife-only discordance, where the woman reports use and the man does not, rose from 7.5% in 2013 to a peak of 15.1% in 2018 before easing to 14.0% in 2024 nationally, and in Ogun State rose from 17.1% to 28.4% over the same period. The decline is thus not diffuse measurement noise but the signature of women adopting methods ahead of, or apart from, their partners. Formally attributing the 8.1-point fall confirms the mechanism: the shrinking share of concordant non-use accounts for almost the entire decline (it fell 8.0 points, ≈ 99% of the total), while genuine mutual use was flat; on the discordance side, wife-only discordance supplied ≈82% of the offsetting rise, with husband-only and method-discordant couples contributing roughly 9% each. Table 2 reports the partition.

**Table 2:** Concordance-pattern decomposition by survey round, national (design-weighted, % of couples).

Pattern	2013	2018	2024	Overall
Concordant active users (both report the same method)	4.1	2.5	4.0	3.5
Wife uses, husband reports none (wife-only discordant)	7.5	15.1	14.0	12.0
Husband reports use, wife none (husband-only discordant)	8.0	7.1	8.7	7.9
Both use, different methods (discordant)	3.0	2.6	3.7	3.1
Concordant non-users (neither uses any method)	77.5	72.7	69.5	73.6

Notes. Practice concordance is the sum of concordant active users and concordant non-users (81.6% in 2013, 75.1% in 2018, and 73.5% in 2024). The fall in agreement is concentrated in wife-only discordance. Component percentages are rounded to one decimal place, so the two concordant cells may not sum exactly to the stated concordance figure and columns may not total 100.

### Correlates of concordance: the inverse gradient

The social patterning of concordance ran opposite to the usual expectation. Bivariately and after adjustment, agreement was highest among the poorest, least-educated, and rural couples and lowest among the wealthier, better-educated, and urban couples, an inverse socioeconomic gradient. Because the dominant form of agreement was shared non-use, this is what the gradient reflects: the couples least exposed to contraceptive uptake had the least to disagree about. In the multivariable logistic model, which adjusted for residence, education, household wealth, educational homogamy, age, and parity, spousal communication carried a negative and significant association with concordance, with an adjusted odds ratio of 0.83. Couples who talked about family planning were less likely, not more, to be classified as concordant, because communication accompanies the asymmetric adoption (wife-only use) that the concordance measure scores as disagreement. Educational homogamy, often assumed to foster agreement, showed no independent association once the other factors were held constant. The unadjusted associations are reported in Table 3, and Table 4 summarises the adjusted model.

**Table 3:** Weighted bivariate association between sociodemographic factors and couple practice concordance, pooled 2013–2024 (N = 23,547).

Factor	Category	N	Concordant %	Wald χ² (df)	p
Residence	Urban	8,708	65.5	271.8 (1)	< 0.001
	Rural	14,839	84.5		
Women’s education	No education	9,965	91.2	553.7 (3)	< 0.001
	Primary	3,953	73.6		
	Secondary	7,330	64.3		
	Higher	2,299	57.9		
Men’s education	No education	7,283	92.1	377.5 (3)	< 0.001
	Primary	4,282	77.9		
	Secondary	7,948	68.8		
	Higher	4,034	63.5		
Wealth quintile	Poorest	5,109	94.0	729.7 (4)	< 0.001
	Poorer	4,802	86.8		
	Middle	4,504	77.2		
	Richer	4,624	66.3		
	Richest	4,508	59.4		
Geopolitical zone	North Central	4,712	80.8	510.5 (5)	< 0.001
	North East	4,479	86.5		
	North West	6,381	86.5		
	South East	2,319	61.3		
	South South	2,673	66.9		
	South West	2,983	56.5		
Women’s age group	15–19	1,726	93.4	267.1 (6)	< 0.001
	20–24	3,958	82.9		
	25–29	5,704	77.6		
	30–34	4,824	74.2		
	35–39	3,914	69.9		
	40–44	2,240	70.7		
	45–49	1,181	74.9		
Parity	No children	2,017	95.3	292.3 (3)	< 0.001
	1–2	7,860	77.7		
	3–4	7,412	71.5		
	5+	6,258	76.6		
Discussed FP (past year)	Discussed	9,214	70.2	179.6 (1)	< 0.001
	Not discussed	14,230	81.5		
Educational homogamy	Same level	13,854	77.5	2.4 (1)	0.122
	Different level	9,693	76.4		

Design-weighted concordance percentages; N denominators unweighted. χ2 statistics are from design-based, PSU cluster-robust Wald tests (degrees of freedom in parentheses). The inverse socioeconomic gradient (concordance highest among the poorest, least-educated and rural couples) reflects near-universal shared non-use among less advantaged couples. Educational homogamy was the only factor not significantly associated with concordance. The family-planning-communication rows total 23,444 rather than 23,547 because 103 couples lack a valid response on the communication item. FP = family planning.

**Table 4:** Multivariable logistic regression of couple practice concordance: pooled and year-stratified adjusted odds ratios (95% CI).

Variable	Pooled aOR	2013	2018	2024
Urban (ref: rural)	0.87 (0.77–0.97)*	0.95 (0.74–1.22)	0.76 (0.63–0.90)**	0.93 (0.78–1.11)
Primary education	0.40 (0.33–0.47)**	0.48 (0.33–0.70)**	0.33 (0.26–0.41)**	0.43 (0.33–0.56)**
Secondary education	0.31 (0.25–0.38)**	0.35 (0.23–0.54)**	0.28 (0.21–0.39)**	0.31 (0.25–0.39)**
Higher education	0.28 (0.22–0.35)**	0.40 (0.25–0.63)**	0.27 (0.18–0.39)**	0.21 (0.16–0.29)**
Poorer	0.52 (0.41–0.64)**	0.30 (0.19–0.49)**	0.60 (0.43–0.83)**	0.68 (0.49–0.94)*
Middle	0.37 (0.30–0.47)**	0.21 (0.12–0.36)**	0.46 (0.34–0.62)**	0.47 (0.34–0.66)**
Richer	0.28 (0.22–0.35)**	0.15 (0.09–0.25)**	0.33 (0.23–0.48)**	0.39 (0.28–0.55)**
Richest	0.24 (0.19–0.31)**	0.13 (0.08–0.23)**	0.26 (0.18–0.38)**	0.37 (0.26–0.53)**
Discussed FP (past year)	0.83 (0.76–0.91)**	0.98 (0.83–1.15)	0.77 (0.66–0.90)**	0.76 (0.66–0.88)**
Educational homogamy	0.95 (0.87–1.04)	0.85 (0.72–1.00)	1.04 (0.89–1.21)	0.99 (0.86–1.15)
Women's age (per year)	1.00 (1.00–1.01)	0.99 (0.97–1.00)	1.01 (1.00–1.03)*	1.00 (0.99–1.02)
Parity (per category)	0.67 (0.63–0.70)**	0.74 (0.67–0.81)**	0.64 (0.59–0.70)**	0.62 (0.57–0.68)**
Survey year 2018 (ref: 2013)	0.81 (0.69–0.95)**	n/a	n/a	n/a
Survey year 2024 (ref: 2013)	0.72 (0.61–0.84)**	n/a	n/a	n/a
N (complete cases)	23,444	8,601	8,045	6,798

Notes. aOR, adjusted odds ratio; CI, confidence interval. Reference categories are rural residence, no education, poorest wealth quintile, no family planning discussion in the past year, and educational heterogamy; the pooled model also adjusts for survey round. Year-specific columns are from logistic models fit separately within each survey round; the pooled column additionally adjusts for survey round. Estimates use the full survey design with PSU cluster-robust inference. * p < 0.05; ** p < 0.01.

The sensitivity analysis confirmed this interpretation: among couples in which at least one partner used a method, communication was positively associated with concordant active use (aOR 1.42, 95% CI 1.17–1.72). In the full sample, communication independently predicted mutual active use (aOR 1.63, 95% CI 1.35–1.97) as well as wife-only discordance (aOR 1.24, 95% CI 1.10–1.39). Thus, the negative pooled association reflects the composition of the outcome—where shared non-use dominates—rather than a detrimental effect of communication itself. Full sensitivity models are reported in S1 Table. Because couples surveyed in 2024 were more urban and better educated than those in 2013, we tested whether the decline was merely compositional. Standardising to the pooled covariate distribution (predictive margins from the adjusted model), concordance still fell from 79.9% in 2013 to 74.9% in 2024, a 5.0-point secular decline holding residence, education, wealth, communication, age and parity constant. Roughly three-fifths of the crude 8.1-point fall is therefore a genuine within-composition decline rather than an artefact of the changing profile of couples surveyed, consistent with the adjusted 2024-versus-2013 odds ratio of 0.72 (Table 4).

Importantly, the negative association between spousal communication and overall concordance should not be interpreted as evidence that communication reduces contraceptive alignment. Because concordance was dominated by shared non-use, communication was associated with couples moving out of this agreement state, into both mutual active use and wife-only use.

### Geography of concordance

Concordance varied sharply across the country and followed the familiar north-south gradient in reproductive behaviour (Fig 2). State-level estimates ranged from about 36% in Ekiti to about 91.5% in Niger, with the high-concordance states concentrated in the far north and the low-concordance states, including Lagos (about 42%) and Ogun (about 46%), in the south. Read together with the decomposition, the map is a map of non-use: the northern states where almost no couples use a method record near-universal agreement, while the southern states where uptake is further along, and asymmetric, record the lowest agreement. The spatial pattern therefore reinforces rather than complicates the national reading. State-level cells are smaller than national ones, so these estimates are read as descriptive of spatial pattern; estimates for all 36 states and the Federal Capital Territory, with cell sizes, are provided in S2 Table.

**Fig 2:**
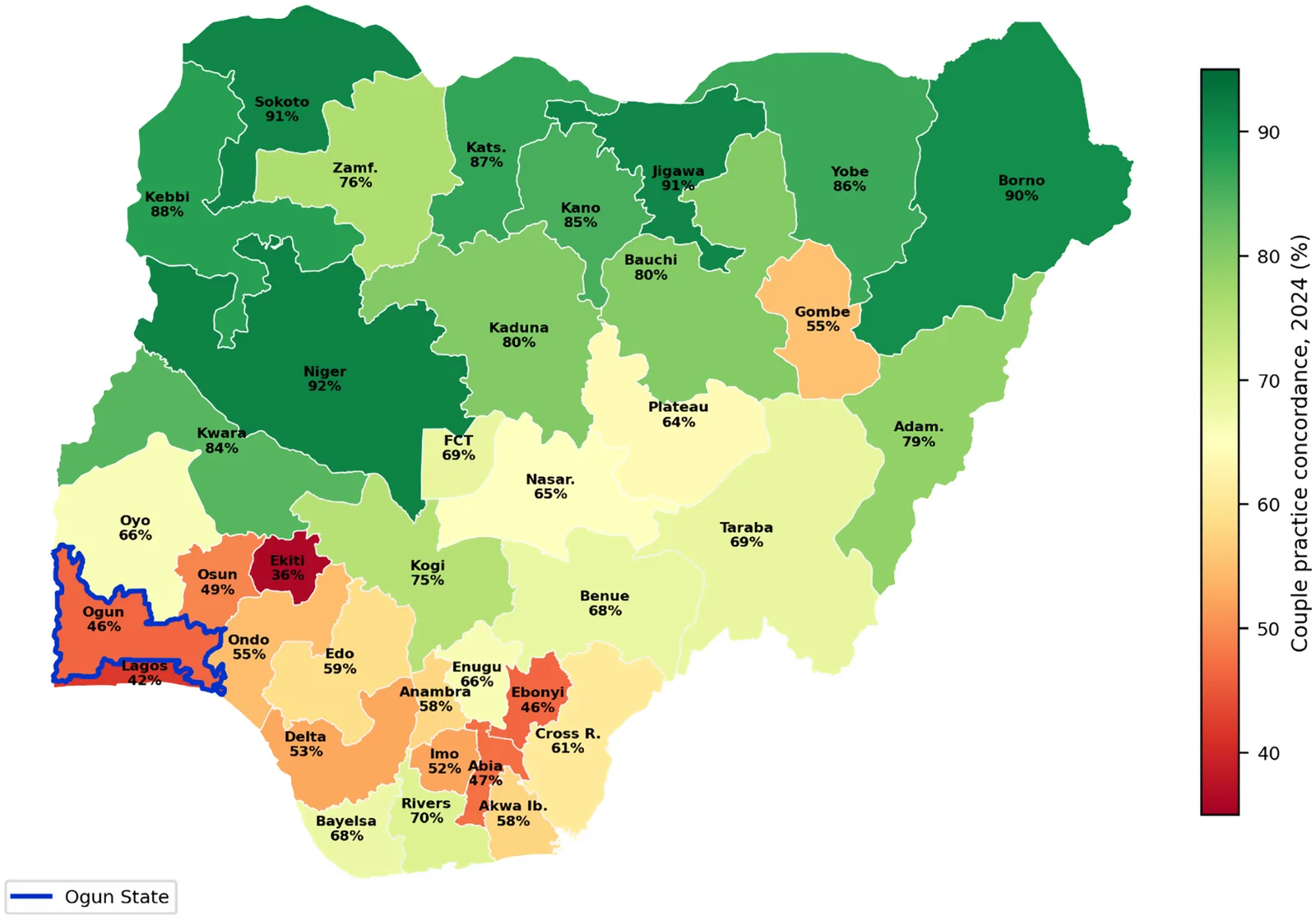
State-level couple practice concordance, Nigeria, 2024 (design-weighted). (a) The Fig 2 map was created by the authors in Python (geopandas/matplotlib). No proprietary or web basemap (e.g., Google Maps) was used. The only base layer is the Nigeria state (admin level 1) boundary shapefile from the “Nigeria – Subnational Administrative Boundaries” dataset. This dataset was produced by the Office of the Surveyor General of the Federation (OSGOF) and UN OCHA and is available at: https://data.humdata.org/dataset/cod-ab-nga (b) The dataset is published under the Creative Commons Attribution for Intergovernmental Organisations licence (CC BY-IGO 3.0): This is available at: https://creativecommons.org/licenses/by/3.0/igo/legalcode. This is an attribution-only licence with no non-commercial or no-derivatives restrictions.

### Ogun State as an acute case

Ogun State concentrates the national pattern in a faster and more visible form. Concordance there fell from 70.0% in 2013 to 60.9% in 2018 and to 46.4% in 2024, so that by the latest round fewer than half of couples agreed on practice, even as spousal communication in the state reached 56.1% (Table 5). Wife-only use rose sharply to 28.1% by 2018 and held near that level (28.4%) in 2024, the same asymmetric-adoption signature seen nationally but steeper. The 2024 round also shows husband-only discordance and method-discordance both roughly doubling to tripling (5.3% to 11.2%, and 5.6% to 14.0%, respectively; Table 5); with only 118 couples in that round, these later swings are read as indicative rather than confirmed shifts. Ogun is not an outlier producing a different phenomenon; it is a leading edge of the same one, which is why it was chosen for the qualitative work.

**Table 5:** Ogun State compared with the national sample, by survey round (design-weighted).

Indicator	Ogun 2013	Nat 2013	Ogun 2018	Nat 2018	Ogun 2024	Nat 2024
N (couples)	124	8,658	195	8,061	118	6,828
Practice concordance, %	70.0	81.6	60.9	75.1	46.4	73.5
Modern use, women, %	29.1	9.7	21.3	14.4	35.9	16.1
FP communication, %	47.4	33.0	48.0	39.0	56.1	42.1
Unmet need, %	23.5	16.1	17.8	17.7	20.4	21.3
Concordant active users, %	10.3	4.1	4.9	2.5	7.2	4.0
Wife-only discordant, %	17.1	7.5	28.1	15.1	28.4	14.0
Husband-only discordant, %	8.4	8.0	5.3	7.1	11.2	8.7
Method-discordant (both use, different methods), %	4.5	3.0	5.6	2.6	14.0	3.7
Concordant non-use, %	59.7	77.5	56.0	72.7	39.2	69.5

Notes. Nat, national. Ogun State cell sizes are small (124, 195, and 118 couples across the three rounds) and are read as indicative rather than precise. The state combines higher and rising female modern use with sharply falling concordant non-use, the same pattern seen nationally in more acute form; the five pattern categories sum to 100 within each column.

### Qualitative corroboration of the mechanism

The qualitative study of 42 couples (84 individuals) across five Ogun local government areas was purposively recruited toward concordant, engaged couples, and its role here is to illuminate mechanism rather than to test prevalence. Most couples were engaged with family planning: 38 of 42 (90.5%) reported having discussed it and 4 had not. Reported current use was high: 29 wives (69.0%) and 29 husbands (69.0%) were currently using a modern method (most often injectables, implants, pills or condoms), 3 wives a traditional method, and 10 couples none. Consistent with the recruitment strategy, the sample was overwhelmingly concordant: 30 couples (71.4%) were concordant users, 10 (23.8%) concordant non-users, and 2 (4.8%) discordant. The sample therefore does not reproduce the population-level wife-only discordance in prevalence terms (nor is it designed to), but the interviews document the relational mechanism behind that population signal: female-led information-seeking and initiation, discussion that settled desired family size without aligning method practice, and, in some couples, independent or covert adoption (one wife described starting a method without telling her husband, who learned of it only later). These qualitative patterns, drawn from the dyadic interview data, are consistent with and help explain the quantitative decomposition reported above.

## Discussion

Across three national rounds, couple practice concordance in Nigeria fell while contraceptive use, spousal communication, and unmet need all rose. The decomposition explains the apparent contradiction. Agreement in this setting was overwhelmingly a shared absence of use, genuine mutual active use never exceeded about 4% of couples, and the decline in concordance was driven by women adopting methods while their partners did not. The concordance figure was falling not because couples were drifting apart in some general sense, but because more women were using contraception in unions where the man was not, a state the standard measure scores as disagreement. What looks like deterioration on the agreement metric is, at least in part, the visible trace of women moving first.

We interpret this pattern as the signature of an incomplete gender transition, and we frame the interpretation associationally because the data are repeated cross-sections and cannot follow individual couples. The Theory of Planned Behaviour locates the proximate driver of use in intentions shaped by attitudes, norms, and perceived control [8]; the Gender and Power framework explains why those elements are distributed unequally within unions, so that women's contraceptive agency can advance while men's involvement lags [9,10]; and the Social-Ecological Model situates both inside the household, community, and policy levels that the north-south gradient makes visible [11]. Together they suggest a transition in which women's practice has begun to change ahead of the couple-level norms and male engagement that would convert wife-only use into mutual, jointly owned use. On that reading, falling concordance is a stage of progress that is incomplete, not a sign that programmes are failing.

The rarity and flatness of mutual active use is the most pointed finding. If couple agreement signified joint protection, the jointly protected category would have grown as national use climbed; instead it sat near 4% throughout. This is consistent with other work showing that male partners in the region often remain peripheral to contraceptive practice even where women's use is rising [15–17]. It also cautions against treating any single-round agreement statistic as evidence of shared decision-making, the use to which concordance is frequently put [1,7].

The inverse socioeconomic gradient sharpens the point. Concordance was highest where uptake was lowest, among the poorest, least-educated, and rural couples, and the negative adjusted association between spousal communication and concordance (odds ratio 0.83) runs the same way: couples who talk about family planning are more likely to be in the asymmetric, wife-led state that the measure records as discordance. Communication itself is not associated with poorer contraceptive agreement. Rather, communication often accompanies contraceptive adoption, and among couples already using a method, communication is positively associated with concordance. The negative overall association is therefore largely driven by the dominance of couples who were not using contraception, which can create a misleading “concordance paradox” in the pooled analysis. Studies that have linked communication to concordance in single cross-sections [18,19] may be capturing settings or stages where mutual use, rather than wife-led adoption, predominates.

Our results align with and extend prior Nigerian and regional work. Cross-sectional analyses have documented substantial spousal disagreement and the salience of male attitudes [4–6], and comparative analyses have flagged the limits of agreement as an outcome [2]. What multi-round, couple-level decomposition adds is the dynamic: it shows that the agreement category is not static, that its internal composition is shifting toward wife-led discordance, and that this shift, rather than any fall in joint use, drives the headline decline. It also reframes a result that earlier single-round studies could only describe as cross-sectional variation [20–22].

The programme implications follow from the mechanism. If falling concordance reflects women adopting ahead of their partners, the response is not to slow women's uptake but to help men catch up, through couple-oriented counselling and male-involvement strategies that turn wife-only use into jointly owned use without making women's access contingent on male approval [15,23,24]. Services should also expect, and not be alarmed by, declining agreement statistics during periods of female-led uptake, and should monitor the composition of concordance, the share that is mutual active use, rather than its headline level. Counselling that supports disclosure where wanted, while protecting women's autonomy and the option of discreet use, is the practical bridge between the wife-only and mutual-use states [3,17].

### Strengths and limitations

The study's strengths are its scale and design. It draws on three rounds of the largest nationally representative couple dataset for Nigeria, uses partners’ independent reports rather than one spouse's account of the other, applies full survey-design estimation with cluster-robust inference, and decomposes the agreement category so that the mechanism behind the trend, not only its direction, is visible. The qualitative study in Ogun State, though recruited toward concordant couples, illuminates the relational mechanism (female-led, sometimes covert adoption) behind the population trend, adding convergent support from a different method.

The limitations bear directly on interpretation. The design is a series of repeated cross-sections, not a panel, so it cannot track change within individual couples; all statements about movement refer to the population, and we have deliberately avoided language implying within-couple trajectories. Method status was self-reported independently by each partner and is therefore subject to both recall and social-desirability bias. Respondents may have difficulty accurately recalling current contraceptive use, while concerns about disclosure, partner disagreement, or perceived social expectations may influence reporting, particularly for methods used without the partner's knowledge. Such biases may operate differently for husbands and wives and may vary across survey rounds, potentially producing either over- or under-estimation of discordance and affecting observed trends in concordance. In particular, covert contraceptive use may be missed when women do not disclose their use, meaning that wife-only discordance could be underestimated. Consequently, the observed discordance should be interpreted as a measure of reported rather than necessarily actual contraceptive practice.

An additional limitation concerns the operational definition of concordance. We defined concordance from partners’ reported current contraceptive use status rather than requiring agreement on the specific contraceptive method. This approach is advantageous for examining whether contraceptive practice is shared or asymmetric and for identifying the transition from shared non-use to active use. However, different definitions of concordance can yield different substantive interpretations. A method-specific definition would provide greater information about whether partners use the same method and would distinguish couples using different methods more explicitly, whereas the present definition prioritises agreement in practice status. Thus, mutual active use in this study should not be interpreted as necessarily representing use of the same method, identical contraceptive effectiveness, or fully shared decision-making. Conversely, retaining shared non-use within the concordance measure allows the study to demonstrate that a high headline concordance can coexist with very little mutual contraceptive use. We therefore interpret the overall concordance measure alongside its component categories rather than as a standalone indicator of successful joint contraceptive practice. The sensitivity analyses further demonstrate that the observed association with spousal communication changes when the non-use-dominated denominator is restricted, supporting caution in interpreting the headline measure.

Finally, the qualitative sample was purposive and concentrated in one southern state; consequently, the mechanisms identified in Ogun State should not be assumed to represent couples in northern Nigeria, where cultural, religious, and gender norms may differ substantially. The qualitative findings therefore serve as contextual and mechanistic corroboration of the quantitative results rather than as nationally generalisable evidence. The incomplete gender transition is offered as an interpretive frame consistent with the cross-sectional pattern, not as a demonstrated trajectory; the repeated cross-section design cannot adjudicate it against alternatives such as cohort or period effects or differential reporting, and establishing it would require panel data that follow the same couples over time.

## Conclusions

Between 2013 and 2024, couple agreement on contraception in Nigeria fell even as use, communication, and unmet need rose, and the decomposition shows why: agreement was mostly shared non-use, joint active use stayed rare, and the decline was driven by women adopting methods ahead of their partners. Concordance, treated as a marker of programme success, behaved instead as a marker of how little was happening, and it fell precisely where contraceptive practice was advancing. The pattern is consistent at the population level with an incomplete gender transition rather than with programme failure.

For research and practice, the implication is to stop reading headline agreement as joint protection, to monitor the composition of concordance rather than its level, and to invest in couple-oriented and male-involvement approaches that can convert women's gains into shared, jointly owned use without making those gains conditional on male approval.

## Supporting information

S1 Table Sensitivity of the spousal-communication–concordance association to the non-use-dominated outcome. Four design-weighted logistic models showing that the negative pooled association is a composition effect.
(DOCX)

S2 Table State-level couple practice concordance, Nigeria 2024 (design-weighted). All 36 states and the Federal Capital Territory with unweighted couple counts.
(DOCX)

S1 File STROBE Checklist. (DOCX)
