# Supplementary material for: Rising contraceptive use, falling couple concordance: A mixed-methods analysis of the concordance paradox in Nigeria
Source: PLOS Glob Public Health. 2026 Oct 30;6(10):e0007450. doi: 10.1371/journal.pgph.0007450 (PMC13722185; doi:10.1371/journal.pgph.0007450)
Supplement: Supplementary file 1 [file pgph.0007450.s001.docx]

**S1 Table.** Sensitivity of the spousal-communication–concordance association to the non-use-dominated outcome (design-weighted logistic regression, primary sampling unit (PSU) cluster-robust; adjusted for residence, education, wealth, educational homogamy, age, parity, and survey round).

| **Model** | **Outcome** | **Sample** | **Communication aOR (95% CI)** | **p** |
| --- | --- | --- | --- | --- |
| A (primary) | Practice concordance (shared non-use + mutual use) | All couples (n = 23,444) | 0.83 (0.76–0.91) | < 0.001 |
| B | Concordant active use vs discordant | Active couples, ≥1 partner uses (n = 6,177) | 1.42 (1.17–1.72) | < 0.001 |
| C | Concordant active use vs all else | All couples (n = 23,444) | 1.63 (1.35–1.97) | < 0.001 |
| D | Wife-only discordance | All couples (n = 23,444) | 1.24 (1.10–1.39) | < 0.001 |

Notes. The primary negative association (Model A) reverses among couples who use a method (Model B) and communication positively predicts genuine mutual active use (Model C), indicating that the pooled negative estimate is a composition effect of an agreement measure dominated by shared non-use, not evidence that communication reduces couple alignment. aOR, adjusted odds ratio. n = 23,444 analytic sample after excluding couples with missing model covariates (99.6% of 23,547 matched couples retained). Source: Nigeria DHS Couples’ Recode 2013–2024, design-weighted (V005; strata V022; PSU V021).
