# Supplementary material for: Rising contraceptive use, falling couple concordance: A mixed-methods analysis of the concordance paradox in Nigeria
Source: PLOS Glob Public Health. 2026 Oct 30;6(10):e0007450. doi: 10.1371/journal.pgph.0007450 (PMC13722185; doi:10.1371/journal.pgph.0007450)
Supplement: Supplementary file 2 [file pgph.0007450.s002.docx]

**S2 Table.** State-level couple practice concordance, Nigeria 2024 (design-weighted; V005 weight, V022 strata, V021 PSU).

| **State** | **Geopolitical zone** | **Practice concordance 2024 (%, design-weighted)** | **Couples (unweighted n)** |
| --- | --- | --- | --- |
| Benue | North Central | 68.3 | 145 |
| FCT | North Central | 68.7 | 150 |
| Kogi | North Central | 75.0 | 203 |
| Kwara | North Central | 84.0 | 205 |
| Nasarawa | North Central | 65.1 | 271 |
| Niger | North Central | 91.5 | 233 |
| Plateau | North Central | 63.9 | 222 |
| Adamawa | North East | 78.6 | 195 |
| Bauchi | North East | 80.1 | 229 |
| Borno | North East | 90.2 | 147 |
| Gombe | North East | 55.2 | 250 |
| Taraba | North East | 68.7 | 139 |
| Yobe | North East | 86.2 | 226 |
| Jigawa | North West | 91.1 | 224 |
| Kaduna | North West | 80.4 | 311 |
| Kano | North West | 85.3 | 309 |
| Katsina | North West | 86.8 | 227 |
| Kebbi | North West | 87.9 | 261 |
| Sokoto | North West | 91.1 | 313 |
| Zamfara | North West | 75.9 | 204 |
| Abia | South East | 47.3 | 110 |
| Anambra | South East | 57.9 | 189 |
| Ebonyi | South East | 46.5 | 214 |
| Enugu | South East | 66.3 | 146 |
| Imo | South East | 52.3 | 202 |
| Akwa Ibom | South South | 57.6 | 130 |
| Bayelsa | South South | 67.8 | 124 |
| Cross River | South South | 60.9 | 125 |
| Delta | South South | 52.6 | 113 |
| Edo | South South | 58.7 | 141 |
| Rivers | South South | 70.3 | 204 |
| Ekiti | South West | 36.1 | 77 |
| Lagos | South West | 41.8 | 140 |
| Ogun | South West | 46.4 | 118 |
| Ondo | South West | 54.9 | 109 |
| Osun | South West | 49.2 | 92 |
| Oyo | South West | 66.0 | 130 |

Notes. Estimates are design-weighted point values; state cells are smaller than national cells and are read as descriptive of spatial pattern rather than as precise point values for any single state. Range: Ekiti 36.1% (lowest) to Niger 91.5% (highest); Lagos 41.8%, Jigawa 91.1%, Ogun 46.4%. The national couple-level practice concordance estimate (73.5% in 2024) is reported in the main text; this table does not include a national row. Source: Nigeria DHS Couples’ Recode 2024.
