# Supplementary material for: Rising contraceptive use, falling couple concordance: A mixed-methods analysis of the concordance paradox in Nigeria
Source: PLOS Glob Public Health. 2026 Oct 30;6(10):e0007450. doi: 10.1371/journal.pgph.0007450 (PMC13722185; doi:10.1371/journal.pgph.0007450)
Supplement: Supplementary file 3 [file pgph.0007450.s003.docx]

**S3 File.** STROBE Statement — checklist of items that should be included in reports of cross-sectional studies.

*Manuscript: Rising contraceptive use, falling couple concordance: a mixed-methods analysis of the concordance paradox in Nigeria, 2013 to 2024. Page and line numbers refer to the double-spaced, line-numbered submission file.*

| **Section / Topic** | **Item** | **Recommendation** | **How addressed in this manuscript** | **Location** |
| --- | --- | --- | --- | --- |
| Title and abstract | 1 | (a) Indicate the study's design with a commonly used term in the title or the abstract | Title: “mixed-methods analysis”; Abstract identifies three rounds of repeated cross-sectional DHS Couples’ Recode data | Title page; Abstract |
|  |  | (b) Provide in the abstract an informative and balanced summary of what was done and what was found | Abstract reports design, sample (23,547 couples), main results and conclusion | Abstract |
| **Introduction** |  |  |  |  |
| Background/rationale | 2 | Explain the scientific background and rationale for the investigation being reported | Concordance treated as a marker of programme success; assumption that agreement and uptake move together is untested | Introduction, paras 1–3 |
| Objectives | 3 | State specific objectives, including any prespecified hypotheses | To establish whether couple concordance rose or fell alongside contraceptive use in Nigeria 2013–2024 and to characterise what that movement reveals | Introduction, final para |
| **Methods** |  |  |  |  |
| Study design | 4 | Present key elements of study design early in the paper | Repeated cross-sectional analysis of three DHS rounds; stated explicitly and repeated in limitations | Methods — Data and study sample |
| Setting | 5 | Describe the setting, locations, and relevant dates, including periods of recruitment, exposure, follow-up, and data collection | Nigeria, nationally representative; DHS rounds 2013, 2018 and 2023–24; qualitative fieldwork in five Ogun State LGAs | Methods — Data and study sample; Qualitative corroboration |
| Participants | 6 | (a) Give the eligibility criteria, and the sources and methods of selection of participants | Couples’ Recode links co-resident married or cohabiting partners each interviewed independently | Methods — Data and study sample |
| Variables | 7 | Clearly define all outcomes, exposures, predictors, potential confounders, and effect modifiers. Give diagnostic criteria, if applicable | Practice concordance defined from independently reported current method status (V312/MV312) and decomposed into five mutually exclusive categories; covariates listed | Methods — Outcome; Statistical analysis |
| Data sources/ measurement | 8 | For each variable of interest, give sources of data and details of methods of assessment (measurement). Describe comparability of assessment methods if there is more than one group | DHS standard questionnaires; partners interviewed separately, so reports are independent and comparably measured | Methods — Data and study sample; Outcome |
| Bias | 9 | Describe any efforts to address potential sources of bias | Both-partner method status available for all 23,547 couples; covariate standardisation tested compositional change; covert use acknowledged as conservative bias | Methods — Statistical analysis; Results; Strengths and limitations |
| Study size | 10 | Explain how the study size was arrived at | All linked couples in the three rounds were included; no sampling beyond the DHS design | Methods — Data and study sample |
| Quantitative variables | 11 | Explain how quantitative variables were handled in the analyses. If applicable, describe which groupings were chosen and why | Age and parity entered continuously/ordinally; education, wealth and zone categorical with stated reference groups; homogamy binary (limitation noted) | Methods — Statistical analysis; Table 4 notes |
| Statistical methods | 12 | (a) Describe all statistical methods, including those used to control for confounding | Design-based descriptive estimation and multivariable logistic regression adjusting for residence, education, wealth, homogamy, age, parity and communication | Methods — Statistical analysis |
|  |  | (b) Describe any methods used to examine subgroups and interactions | Year-stratified models fitted separately within each round; Ogun State analysed as a focal case | Methods — Statistical analysis; Table 4 |
|  |  | (c) Explain how missing data were addressed | Outcome complete for all 23,547 couples; 103 couples (0.4%) excluded from adjusted models for missing spousal-communication response | Methods — Data and study sample; Results |
|  |  | (d) If applicable, describe analytical methods taking account of sampling strategy | Women’s weight V005/1,000,000, strata V022, PSU V021, Taylor-series linearisation with cluster-robust standard errors; round indicators in pooled models | Methods — Survey design and estimation |
|  |  | (e) Describe any sensitivity analyses | Model restricted to couples with ≥1 partner using; models for mutual active use and for wife-only discordance; covariate standardisation of the trend | Results; S1 Table |
| **Results** |  |  |  |  |
| Participants | 13 | (a) Report numbers of individuals at each stage of study | 8,658 (2013), 8,061 (2018) and 6,828 (2024) couples; pooled 23,547; 23,444 complete cases | Results — Sample characteristics |
|  |  | (b) Give reasons for non-participation at each stage | Only exclusion was 103 couples lacking a valid spousal-communication response | Results — Sample characteristics |
|  |  | (c) Consider use of a flow diagram | Not used; exclusions are a single step affecting 0.4% and are described in text | Results — Sample characteristics |
| Descriptive data | 14 | (a) Give characteristics of study participants and information on exposures and potential confounders | Age, residence, education of both partners, wealth, living children and educational homogamy by round | Table 1 |
|  |  | (b) Indicate number of participants with missing data for each variable of interest | 103 couples missing the communication item; all other analysis variables complete | Results — Sample characteristics; Table 3 notes |
| Outcome data | 15 | Report numbers of outcome events or summary measures | Concordance 81.6%, 75.1% and 73.5% across rounds; five-category decomposition reported for each round | Table 2; Figure 1 |
| Main results | 16 | (a) Give unadjusted estimates and, if applicable, confounder-adjusted estimates and their precision. Make clear which confounders were adjusted for and why included | Unadjusted associations in Table 3; adjusted odds ratios with 95% CIs in Table 4; reference categories and adjustment set stated in the table notes | Tables 3 and 4 |
|  |  | (b) Report category boundaries when continuous variables were categorized | Wealth quintiles, education levels, age groups and parity groups defined in Table 3 | Table 3 |
|  |  | (c) If relevant, consider translating estimates of relative risk into absolute risk | Predictive margins reported: standardised concordance 79.9% (2013) to 74.9% (2024) | Results — Correlates of concordance |
| Other analyses | 17 | Report other analyses done—eg analyses of subgroups and interactions, and sensitivity analyses | Year-stratified models; Ogun State case; decomposition attribution of the 8.1-point fall; four sensitivity models | Table 4; Table 5; S1 Table |
| **Discussion** |  |  |  |  |
| Key results | 18 | Summarise key results with reference to study objectives | Concordance fell while use, communication and unmet need rose; agreement was overwhelmingly shared non-use | Discussion, para 1 |
| Limitations | 19 | Discuss limitations of the study, taking into account sources of potential bias or imprecision. Discuss both direction and magnitude of any potential bias | Repeated cross-sections cannot track within-couple change; self-report and social desirability; covert use under-measured (conservative direction stated); small state cells; binary homogamy; purposive qualitative sample | Strengths and limitations |
| Interpretation | 20 | Give a cautious overall interpretation of results considering objectives, limitations, multiplicity of analyses, results from similar studies, and other relevant evidence | Interpreted associationally as an incomplete gender transition, explicitly not as a demonstrated trajectory; set against prior Nigerian and regional work | Discussion, paras 2–7 |
| Generalisability | 21 | Discuss the generalisability (external validity) of the study results | Quantitative findings nationally representative of married and cohabiting couples; qualitative strand purposive and confined to one southern state | Strengths and limitations |
| **Other information** |  |  |  |  |
| Funding | 22 | Give the source of funding and the role of the funders for the present study and, if applicable, for the original study on which the present article is based | The authors received no specific funding for this work (entered in the submission system’s financial disclosure field, per PLOS policy) | Submission system — Financial Disclosure |

Note. An Explanation and Elaboration article discusses each checklist item and gives methodological background and published examples of transparent reporting. The STROBE checklist is best used in conjunction with that article, freely available at https://www.strobe-statement.org. Item 22 (Funding) is reported in the submission system rather than the manuscript file, in line with PLOS policy that funding information must not appear in the manuscript.
